# Epidemiology and infection control of Methicillin-resistant *Staphylococcus aureus* in a German tertiary neonatal intensive and intermediate care unit: A retrospective study (2013–2020)

**DOI:** 10.1371/journal.pone.0275087

**Published:** 2022-09-21

**Authors:** Carolin Böhne, Leonard Knegendorf, Frank Schwab, Ella Ebadi, Franz-Christoph Bange, Marius Vital, Dirk Schlüter, Gesine Hansen, Sabine Pirr, Corinna Peter, Bettina Bohnhorst, Claas Baier

**Affiliations:** 1 Department of Pediatric Pulmonology, Allergology and Neonatology, Hannover Medical School (MHH), Hannover, Germany; 2 Institute for Medical Microbiology and Hospital Epidemiology, Hannover Medical School (MHH), Hannover, Germany; 3 Institute of Hygiene and Environmental Medicine, Charité - University Medicine, Berlin, Germany; Tribhuvan University, NEPAL

## Abstract

In preterm and term infants who require intermediate or intensive care Methicillin-resistant *Staphylococcus aureus* (MRSA) infection can lead to significant morbidity. In this study MRSA colonization and infection were assessed in a mixed tertiary neonatal intensive and intermediate care unit in Germany over an 8-year period (2013–2020). We investigated patient-related factors, associated with nosocomial MRSA acquisition, and we discuss our infection control concept for MRSA. Of 3488 patients treated during the study period, 24 were MRSA positive patients, corresponding to 26 patient hospital stays. The incidence was 0.7 MRSA patients per 100 patients. The incidence density was 0.4 MRSA patient hospital stays per 1000 patient days. Twelve patients (50%) acquired MRSA in the hospital. One patient developed a hospital acquired MRSA bloodstream infection 9 days after birth (i.e., 0.03% of all patients on the ward during the study period). A total of 122 patients had to be screened to detect one MRSA positive patient. In a logistic regression model, the use of 3^rd^ generation intravenous cephalosporin (cefotaxim) was associated with nosocomial MRSA acquisition compared with matched control patients who did not acquire MRSA. In sum, the burden of MRSA colonization and infection in the ward was low during the study period. A comprehensive infection control concept that included microbiologic colonization screening, prospective infection surveillance together with isolation and emphasis on basic hygiene measures is essential to handle MRSA in this specialized setting.

## Introduction

In hospitalized preterm or term infants requiring intermediate or intensive care Methicillin-resistant *Staphylococcus aureus* (MRSA) infection can lead to relevant morbidity [1], especially in an endemic situation [2]. Both MRSA colonization [3] and healthcare-associated infection (HAI) occur [4], and in some cases MRSA colonization is followed by MRSA infection, such as bloodstream infection (BSI) [5, 6]. Moreover, MRSA outbreaks can occur in neonatal intermediate and intensive care units [7]. Taken together, the MRSA associated burden of disease is high, resulting in intensive worldwide infection control efforts during the last decades in neonatal care (e.g., [6, 8, 9]). Frequently used measures include active microbiologic MRSA colonization screening, prospective infection surveillance, decolonization approaches, hand hygiene training for staff and barrier precautions for MRSA carriers including spatial isolation (e.g., single room or cohort isolation).

In Germany, colonization screening for MRSA in neonatal intensive care units (NICUs) is publicly recommended and widely implemented [10, 11], including our unit [12]. In addition, prospective HAI surveillance is mandatory in tertiary neonatal intensive care units in Germany [13], and Nation-wide surveillance programs have been installed (e.g., the NEO-KISS surveillance program for preterm infants with a birth weight of less than 1500 g) [14].

In this study, the burden of MRSA colonization and infection was assessed in a tertiary NICU with an intermediate care unit in Germany over an 8-year period. Moreover, we investigated patient-related factors, associated with nosocomial MRSA acquisition, and we discuss our infection control concept for MRSA.

## Methods

### Study type

We conducted a retrospective analysis (2013–2020), of our systematic MRSA colonization and infection surveillance at the tertiary NICU with intermediate care unit of Hannover Medical School, Germany. We identified all patients with MRSA in this period and collected their patient data.

Moreover, we investigated the characteristics of patients with hospital acquired MRSA (cases) and of patients without MRSA (controls). Two controls were matched to one case. The controls were hospitalized at the same time (year, month) as the case. Moreover, the selection of control patients aimed for a comparable gestational age (maximum difference of 22 days) and a comparable “time at risk” (maximum difference of 43 days) in relation to the case. The “time at risk” was defined as days from admission to first MRSA acquisition for the cases and as days from admission to discharge for the control patients.

The ethics committee of the Hannover Medical School approved the study (number 9665_BO_K_2021). Being a retrospective study, the need of informed consent was waived by the ethics committee and the data protection commissioner of Hannover Medical School.

### Setting

The unit provided 10 intensive care beds (located in 3 rooms) and 14 intermediate care beds (located in 5 rooms). The ward was temporarily relocated in 2019/2020 due to renovation. Assigned and specialized nursing staff, physicians and cleaning staff serviced the ward. The unit specialized in the care of extremely and very low birth weight (ELBW and VLBW) preterm infants. In addition, near-term and term infants requiring intensive or intermediate care treatment or monitoring (e.g., postoperative, neonates with respiratory distress syndrome, congenital syndromes or malformations) were also admitted. During the study period, the nursing staff to patient ratio varied from 1:1 to 1:4 depending on the patient’s clinical condition, gestational age and in compliance with national nursing care guidelines.

### Infection control concept

Culture-based MRSA screening was performed for all patients upon admission to the ward. Moreover, patients with an actual weight of less than 1500 g and/or in need of intensive care treatment were rescreened weekly during their stay on the ward. Patients who tested positive for MRSA were strictly isolated in a single room with 1 incubator or bed; however the cohorting of patients was allowed if the respective MRSA antibiograms were similar. Medical equipment was exclusively allocated to the isolation room. Following patient discharge, the patient room and reusable medical equipment were intensively cleaned and disinfected. A decolonization protocol based on mupirocin (nasal mucosa treatment) and octenidine-dihydrochloride (skin treatment) was applied for individual patients. During direct contact with a MRSA patient, healthcare workers wore gowns, gloves and a surgical mask. Healthcare workers were not systematically screened for MRSA carriage but were intensively instructed in standard precautions including hand hygiene. Parents and visitors were also intensively and repeatedly instructed in hygiene measures. As of 2017, hospitalized women at risk of premature delivery were screened for MRSA (nasopharyngeal, rectal and vaginal swabs) in the Department of Obstetrics and Gynecology.

Specialized infection control staff prospectively and actively monitored HAIs and performed audits, training sessions and monitoring on the ward (e.g., hand hygiene compliance observation).

### Data acquisition and definitions

Two of the authors (LK and CB) independently reviewed the microbiologic laboratory information system (m/LAB, Dorner, Müllheim, Germany) and the in-house infection control software to identify all MRSA screening specimens and all MRSA positive specimens with the corresponding patients (study period 2013–2020). Screening numbers were calculated using pandas (v1.2.2) in Python 3.8.

MRSA acquisition occurring on day 3 or later of the stay in the ward without a history of MRSA was defined as hospital acquired (nosocomial). An infection was assumed when MRSA occurred in a microbiologic specimen taken for infection diagnostic purposes (e.g., blood culture), antibiotic treatment was initiated (at least 5 days) and the infection was documented in the patient’s chart. Demographic and clinical information was extracted from the patient charts by another author (CBO). The Centre for Information Management of Hannover Medical School provided the total number of patients, patient hospital stays and patient days in the ward.

### Microbiological diagnostics (MRSA screening)

The microbiological diagnostics were carried out at the ISO 15189 accredited laboratory of the Institute for Medical Microbiology and Hospital Epidemiology at Hannover Medical School.

MRSA screening specimens consisted of a nasopharyngeal and a rectal swab for each patient, supplemented by respiratory secretions for ventilated patients. Screening swabs were cultured on a MRSA selective agar plate (Brilliance MRSA 2 AGAR, Thermo Fisher Scientific, Waltham, USA) and incubated for 22 to 24 hours at 37 °C. In the case of growth, species were identified by a matrix-assisted laser desorption/ionization time of flight mass spectrometry system (bioMérieux, Marcy-l’Étoile, France). In addition, *Staphylococcus aureus* grown on the selective medium was tested using a commercial PBP2a assay (Alere^™^ PBP2a Culture Colony Test, Alere Inc., Scarborough, ME, U.S.A.) to rapidly confirm a possible MRSA diagnosis. Antimicrobial susceptibility was primarily tested with VITEK^®^ 2 (bioMérieux, Marcy-l’Étoile, France) or, alternatively, with the microdilution-based Merlin Micronaut system (Merlin Diagnostika, Bornheim-Hesel, Germany). A *Staphylococcus aureus* isolate was classified as MRSA when two of the following criteria were met: i) growth on selective agar, ii) phenotypic resistance to oxacillin and iii) verification of PBP2a. If a *Staphylococcus aureus* isolate was found in a specimen taken for infection diagnostic purposes, the same criteria for MRSA classification applied. The microbiological examinations were part of the routine diagnostic at the time point when the MRSA isolate was found in the study period.

For the study at hand, we considered the first positive MRSA sample found in the respective patient hospital stay.

### Statistical analysis and visualization

The incidence and incidence density of MRSA were calculated as the number of MRSA patients per 100 patients and MRSA patient hospital stays per 1000 patient days, respectively. We described the entire MRSA cohort stratified by “hospital acquired MRSA” vs. “non-hospital acquired MRSA” and the cohort of the matched case control study stratified by “patients with hospital acquired MRSA” vs. “control patients without MRSA”. For continuous parameters, the results are shown as median with interquartile range and for categorical parameters, they are shown as number and percentage. For categorical parameters, differences were tested with the Chi Square test, and for continuous variables, differences were tested with the Wilcoxon rank sum test. To analyze risk factors for nosocomial MRSA acquisition in the case control study a multivariable analysis was performed using a logistic regression model by stepwise forward variable selection. The significance level was set to 0.05.

All statistical test results were considered significant at p < 0.05. All analyses were exploratory in nature and performed using SPSS 26 (IBM SPSS statistics, Somer, NY, USA) and SAS 9.4 (SAS Institute, Cary, NC, USA).

Antimicrobial susceptibility profiles were manually one-hot encoded and hierarchically clustered using maximum distance and Ward’s linkage using R 4.1.2. Antimicrobial resistance profiles were visualized using the package ggtree (v3.2.1) [15]. Subsequently, Adobe Photoshop (Adobe Inc., Mountain View, CA, USA) was used to remove the dendrogram and rearrange the color legends.

## Results

### Epidemiology and clinical characteristics of MRSA patients

During the 8-year study period between 2013 and 2020, 3488 patients corresponding to 3705 patient hospital stays in the ward were recorded. These patients generated 64249 patient days (i.e., an average length per patient hospital stay of 17.3 days).

Overall, the study identified 24 MRSA patients (2013/14: n = 4; 2015/16: n = 5; 2017/18: n = 5; 2019/20: n = 10) corresponding to 26 MRSA patient hospital stays (2 patients had 2 separate stays on the ward). The incidence was 0.7 MRSA patients per 100 patients (i.e., 0.7% of all treated patients). The incidence density was 0.4 MRSA patient hospital stays per 1000 patient days. Twelve patients had hospital acquired MRSA (50%). In one patient, the mother was a known MRSA carrier. All 24 patients exhibited MRSA colonization (mucosal or skin carriage). The most common colonization sites were nasopharyngeal (in 20 MRSA patient hospital stays, 76.9%) and rectal (in 16 MRSA patient hospital stays, 61.5%).

One patient (gestational age: 37.4 weeks) developed a hospital acquired MRSA bloodstream infection (aerobe blood culture, time to positivity 22 hours and 27 minutes) 9 days after birth (i.e., 0.03% of all patients on the ward in the study period). This infection was associated with an infected peripherally inserted venous catheter (purulent catheter insertion site with MRSA positive swab). The treatment included intravenous vancomycin, local antiseptics and the removal of the catheter. The patient recovered completely. Four patients received a decontamination treatment (mupirocin and octenidine-dihydrochloride), but only one treatment was persistently successful with repetitive MRSA negative swabs during the remaining hospital stay.

Table 1 shows the characteristics of the entire MRSA cohort stratified by acquisition mode (hospital acquired vs. non-hospital acquired). The infants in the hospital acquired MRSA group had a significantly lower gestational age and a significantly longer hospital stay than those in the non-hospital acquired MRSA group.

**Table 1:** Epidemiological and clinical characteristics of the MRSA patients.

Parameter	All MRSA patient hospital stays (A)	Patient hospital stays with hospital acquired MRSA (B)	Patient hospital stays with non-hospital acquired MRSA (C)	p-value* (B vs. C)
Total number of hospital stays	26 (100%)	12 (100%)	14 (100%)	-
Median length of stay in days [IQR]	13 [3–36]	37 [10–50.5]	4 [2–17]	**0.002**
Discharge from ward to home	12 (46.2%)	5 (41.7%)	7 (50%)	0.671
Transfer from ward to another ward	14 (53.8%)	7 (58.3%)	7 (50%)	0.671
Discharge reason death	0 (0%)	0 (0%)	0 (0%)	-
Median birth weight in gram [IQR]	1995 [1220–2910]	1590 [1160–2370]	2462.5 [1795–3000]	0.129
Median gestational age in days (weeks) [IQR]	241.5 (34.5) [219 (31.3)—265 (37.9)]	232 (33.1) [204.5 (29.2)—241.5 (34.5)]	263 (37.6) [240 (34.3)—276 (39.4)]	**0.015**
Female	9 (34.6%)	4 (33.3%)	5 (35.7%)	0.899
Urine positive	0 (0%)	0 (0%)	0 (0%)	-
Rectal positive	16 (61.5%)	8 (66.7%)	8 (57.1%)	0.619
Wound positive	0 (0%)	0 (0%)	0 (0%)	-
Nasopharyngeal positive	20 (76.9%)	10 (83.3%)	10 (71.4%)	0.473
Respiratory secretions positive	3 (11.5%)	0 (0%)	3 (21.4%)	0.088
Blood culture positive	1 (3.8%)	1 (8.3%)	0 (0%)	0.271
Venous catheter tip positive	1 (3.8%)	1 (8.3%)	0 (0%)	0.271
Drainage positive	0 (0%)	0 (0%)	0 (0%)	-
Bile positive	0 (0%)	0 (0%)	0 (0%)	-
Other positive	4 (15.4%)	3 (25%)	1 (7.1%)	0.208
Known MRSA colonization of mother	1 (3.8%)	1 (8.3%)	0 (0%)	0.271
Colonization	26 (100%)	12 (100%)	14 (100%)	-
Infection with MRSA	1 (3.8%)	1 (8.3%)	0 (0%)	0.271

*2 tailed p-value, Chi-square test for categorical parameters and Wilcoxon rank sum test for continuous parameters. Significant results are displayed in bold. IQR = Interquartile range.

### Microbiological characteristics

**Fig 1:**
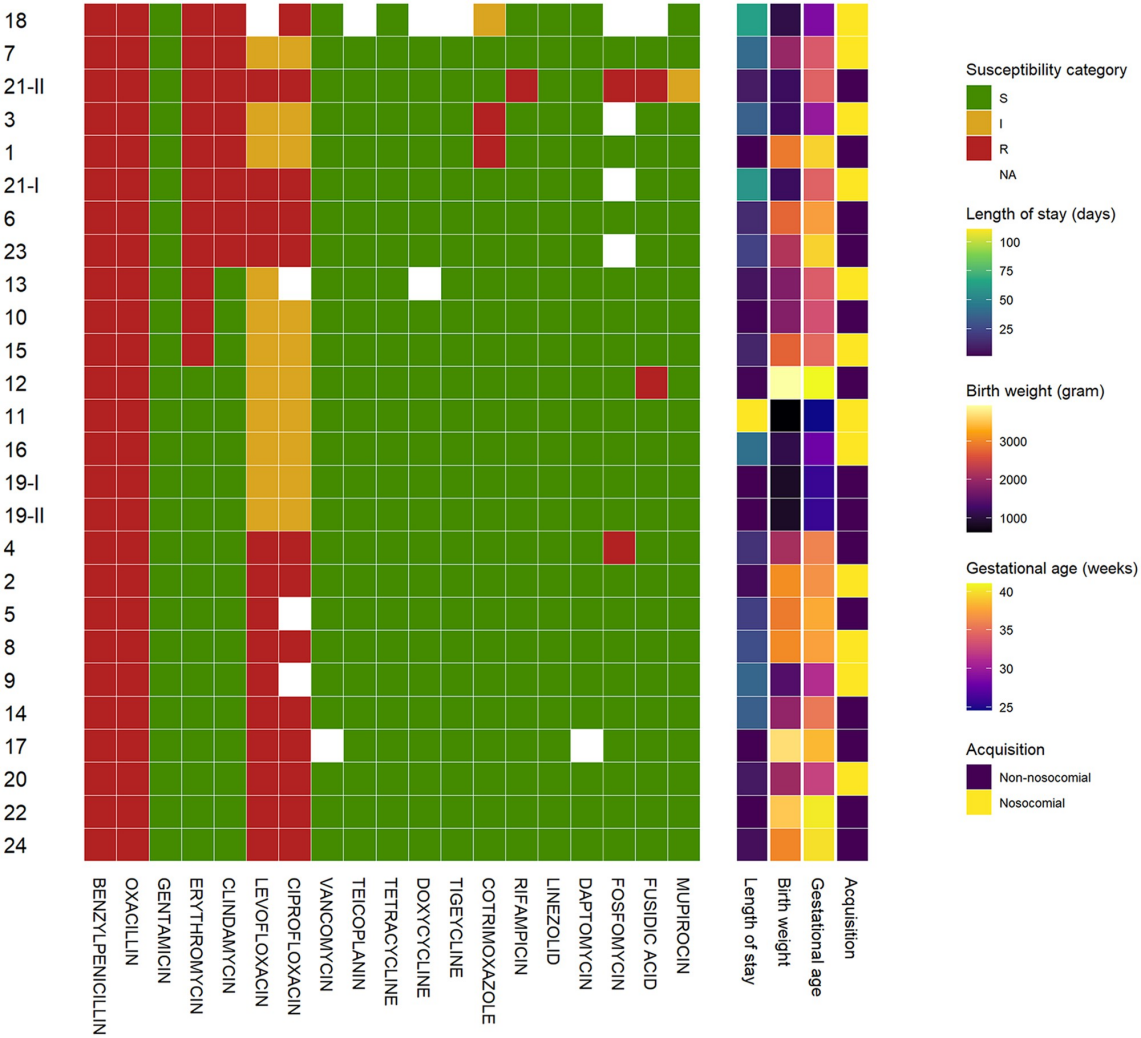
Antibiotic susceptibility pattern of the patients’ MRSA isolates (n = 24 patients, n = 26 MRSA isolates). Arabic numbers indicate patients. For patient 19 and patient 21, two isolates from each two different hospital stays are shown (Roman numbers). Row order is based on the hierarchical clustering of susceptibility patterns using maximum distance and Ward’s linkage.

We evaluated 26 MRSA isolates found in the respective patient hospital stays. In 25 MRSA isolates, resistance was attributable to PBP2a expression verified by immunodiffusion. One MRSA isolate had a negative PBP2a test result, possibly indicating another resistance mechanism. The antimicrobial susceptibility patterns of the various MRSA isolates differed (see Fig 1) and offered adequate potential treatment options including susceptibility to glycopeptides. Fifteen isolates (57.7%) were susceptible to erythromycin, 18 isolates (69.2%) were susceptible to clindamycin and 24 isolates (92.3%) were susceptible to trimethoprim/sulfamethoxazole either under standard or increased exposure. In the two patients with two separate hospital stays, one patient (patient 19) carried MRSA isolates with similar susceptibility; the other patient (patient 21) carried a MRSA isolate with increased resistance during the second stay compared to the one found in the first hospital stay.

### Microbiologic MRSA screening

Overall 2927 patients were screened for MRSA at least once in the study period in the ward (i.e., 83.9% of all patients admitted to the ward). Against the background of 24 MRSA positive patients found among the 2927 patients screened, on average, 122 (2927:24) patients had to be screened to detect one MRSA positive patient. In total, 14261 MRSA screening samples (nasopharyngeal and rectal swabs, respiratory secretions) were obtained (i.e., on average 4.9 screening samples per patient screened).

### Matched case control study of hospital acquired MRSA

Table 2 shows the results of the matched case control study of hospital acquired MRSA (patients with hospital acquired MRSA, n = 12 vs. patients without MRSA acquisition, n = 24). The basic characteristics, including the matching parameters i) gestational age and ii) “time at risk”, were comparable in the two groups (no significant difference between either group; due to limited patient numbers, we accepted a maximum difference of 22 days regarding gestational age and 43 days regarding “time at risk” in individual case/control pairs–see Methods section). Looking at the parameters during the “time at risk”, the presence of a central venous catheter and the use of a 3^rd^ generation intravenous cephalosporin (cefotaxim) were significantly more frequent found among MRSA patients.

**Table 2:** Comparison of patients with hospital acquired MRSA (cases, n = 12) and without MRSA (controls, n = 24)—matched case control study.

Parameter	Patients with hospital acquired MRSA (cases)	Patients without MRSA (controls)	p-value*
**Basic characteristics**			
Total number of patients	12 (100%)	24 (100%)	-
Median length of stay in days [IQR]	37 [10–50.5]	18.5 [8.5–38.5]	0.283
Discharge from ward to home	5 (41.7%)	6 (25%)	0.306
Transfer from ward to another ward	7 (58.3%)	18 (75%)	0.306
Discharge reason death	0 (0%)	0 (0%)	-
Median birth weight in gram [IQR]	1590 [1160–2370]	1755 [1132.5–2192.5]	0.973
Median gestational age in days (weeks) [IQR]	232 (33.1) [204.5 (29.2)—241.5 (34.5)]	226 (32.3) [204.5 (29.2)—238.5 (34.1)]	0.775
Female	4 (33.3%)	9 (37.5%)	0.806
Median time at risk in days [IQR]	15 [6–28.5]	18.5 [8.5–38.5]	0.430
**Parameters during “time at risk”**			
Neutrophil leukocytes <1000/μL	1 (8.3%)	4 (16.7%)	0.496
Hemoglobin <8g/dL	0 (0%)	1 (4.2%)	0.473
Erythrocyte transfusion	3 (25%)	3 (12.5%)	0.343
Thrombocytes <150.000/μL	4 (33.3%)	5 (20.8%)	0.414
Central venous catheter	5 (41.7%)	2 (8.3%)	**0.017**
Vancomycin lock for central venous catheter	1 (8.3%)	0 (0%)	0.151
Peripheral venous catheter	12 (100%)	24 (100%)	-
Invasive ventilation	4 (33.3%)	3 (12.5%)	0.137
Non-invasive ventilation	9 (75%)	17 (70.1%)	0.792
Transurethral catheter	2 (16.7%)	1 (4.2%)	0.201
Surgery	1 (8.3%)	0 (0%)	0.151
Systemic (intravenous) antibiotic application	6 (50%)	9 (37.5%)	0.473
Cefotaxim	5 (41.7%)	1 (4.2%)	**0.004**
Vancomycin	2 (16.7%)	2 (8.3%)	0.453
Meropenem	0 (0%)	2 (8.3%)	0.303
Tobramycin	5 (41.7%)	9 (37.5%)	0.809
Probiotics	2 (16.7%)	5 (20.8%)	0.766

*2 tailed p-value, Chi-square test for categorical parameters and Wilcoxon rank sum test for continuous parameters. Significant results are displayed in bold. IQR = Interquartile range. Two controls were matched to one case. The controls were hospitalized at the same time (year, month) as the case. Moreover, the selection of control patients aimed for comparable gestational age (maximum difference of 22 days) and a comparable “time at risk” (maximum difference of 43 days) in relation to the cases. The “time at risk” was defined as days from admission to first MRSA acquisition for the cases and as days from admission to discharge for the control patients.

In the multivariable analysis by logistic regression, the use of a 3^rd^ generation intravenous cephalosporin (cefotaxim) was independently associated with the hospital acquired MRSA patients compared to the matched patients who did not acquire MRSA (OR 16.4; 95%-CI: 1.6–165.1). Neither different modes of ventilation (invasive or non-invasive), nor the general use of systemic intravenous antibiotics was significantly associated with MRSA acquisition.

## Discussion

In the present study, we retrospectively analyzed MRSA colonization and infection at a tertiary NICU with an intermediate care unit in Germany. During the study period from 2013 to 2020 MRSA was detected in 0.7% of all patients. This rate is comparable to results reported from a NICU in Vancouver, Canada (0.68% MRSA colonization rate, 2010–2014) [16]. In contrast, a study from a Greek neonatal intensive care unit (2014–2018) reported a MRSA rate of 5% [17]. In another study from Germany (2012 and 2013), the MRSA rate was 2.3% among very low birth weight infants [18]. MRSA rates are known to vary globally and temporally due to several reasons, such as varying infection control policies and specific characteristics of prevalent clones [19]. However, Germany has experienced a decline in the MRSA infection burden in hospitals in recent years [20].

With the exception of fluoroquinolones, the MRSA isolates from the present study overall had a rather low non-beta lactam resistance rate matching isolates circulating in the European community [21]. Specifically, a susceptibility rate of 69.2% to clindamycin identified in our study and a susceptibility rate of 81.2% in another European analysis [22] may point to an increased resistance rate to this antibiotic agent.

In our cohort, only one patient had a MRSA infection (0.03% of all patients in the study period), which was a hospital acquired bloodstream infection due to an infected venous catheter. In the Canadian study mentioned above [16], 3 patients developed MRSA bacteremia (0.1% of all admissions). Accordingly, data from the German Neonatal Network (GNN) suggest a MRSA BSI rate of 0.1% resulting in a mortality rate of 6.3% for a high-risk population < 29 weeks of gestation [11]. In sum, this finding indicates that the occurrence of MRSA infection was low in our ward, which corresponds to findings from comparable settings. Comprehensive infection control guidance (e.g., training, audits) might have contributed to this low rate.

Different risk factors are associated with MRSA acquisition: In a matched case control study by Balamohan et al., the authors found high colonization pressure to be the only statistically significant independent risk factor for MRSA acquisition in an endemic setting in a level IV NICU [23]. In a MRSA outbreak at a Danish level II NICU, caesarian section and the application of nasal continuous positive airway pressure have been reported to be independent risk factors for MRSA colonization [24]. Regarding patient-related factors, a systematic review by Washam et al. showed that both low gestational age (<32 weeks) and low birth weight (<1500 g) were associated with MRSA colonization [3]. In our cohort, we identified the use of 3^rd^ generation cephalosporin (cefotaxim) as an independent risk factor for nosocomial MRSA acquisition.

Interestingly, in a study by Bozella et al., the enhanced cleaning of reusable equipment on a NICU, but not systematic MRSA decolonization, led to a reduction of nosocomial MRSA acquisition [25]. This finding underlines that environmental contamination might play a role in MRSA acquisition and needs to be addressed in a comprehensive infection control concept. To this end, we have implemented strict general cleaning and disinfection guidelines in the NICU and specific guidelines for MRSA patient rooms. A recent study by the GNN emphasizes the importance of sepsis surveillance and screening programs in extremely low birth weight infants and underlined the positive effects of antibiotic stewardship programs for the development of sepsis in these high-risk patients [11].

In our study, MRSA acquisition occurred in one case presumably due to a colonized mother. The introduction of MRSA to the NICU by colonized parents has often been discussed. The risk for introduction might vary due to the heterogeneous geographical MRSA burden; for instance, a very low MRSA prevalence among pregnant women was recently reported in Denmark [26].

Eighty-four percent of all admissions in this study were screened at least once for MRSA, which demonstrates good adherence to our screening policy. The vast majority of the 16% of patients who had not been screened, had a short stay on the ward of <48 hours, e.g., for postoperative monitoring.

Our infection control concept for MRSA is based on a culture-based screening program complemented by continuous prospective infection surveillance, a rigorous isolation policy for MRSA positive patients, and hand hygiene training for staff and parents. Regarding the screening approach, the number of patients who needed to be screened to detect one MRSA carrier was 122, which is quite high. However, culture-based screening using a modern selective agar facilitates screening efforts. Nonetheless, we are aware that economic resources for such intensive screening might not be available in all settings. Moreover, during the study period we focused on basic infection control principles, such as hand hygiene and environmental cleaning. To this end, we used repetitive audits and compliance monitoring, but we did not use MRSA screening for healthcare workers.

The analysis at hand has potential limitations and strengths. With respect to the limitations, the number of MRSA patients in this study is rather small (due to the low MRSA burden in Germany and the single center setting), which limits the possibilities for statistical analyses of small effects. Moreover, the multivariable analysis of the matched case control study of hospital acquired MRSA was calculated with a usual logistic regression model because the conditional logistic regression model—the most appropriate method for case control studies—did not converge. All our analyses were exploratory in nature. In addition, we could not evaluate the role of potentially MRSA positive parents in detail, as screening for mothers only started in the last third of the study period and fathers were generally not screened. Regarding strengths, we can provide a very reliable overview of the “true” MRSA burden on our ward due to good screening adherence and rigorous colonization/infection surveillance over a period of 8 years. The good screening adherence is also important for the matched case control study of hospital acquired MRSA, as both groups were similarly screened.

Moreover, we provide practical insights into MRSA infection control in neonatal intensive and intermediate care. In future works, the evaluation of Methicillin-susceptible *Staphylococcus aureus* (MSSA) is also of high interest in neonatal intensive care [27].

In conclusion, the burden of MRSA colonization and infection was low in our ward. We found in a case control approach that 3^rd^ generation cephalosporin use was significantly associated with nosocomial MRSA acquisition. A comprehensive infection control concept, including microbiologic colonization screening and prospective infection surveillance, together with isolation and emphasis on basic hygiene measures was essential to handle MRSA.
